# CO_2_ Capture and *in situ* Catalytic Transformation

**DOI:** 10.3389/fchem.2019.00525

**Published:** 2019-07-24

**Authors:** Hong-Chen Fu, Fei You, Hong-Ru Li, Liang-Nian He

**Affiliations:** ^1^College of Pharmacy, Nankai University, Tianjin, China; ^2^State Key Laboratory and Institute of Elemento-Organic Chemistry, College of Chemistry, Nankai University, Tianjin, China

**Keywords:** CO_2_ capture, activation, conversion, *in situ* catalysis, green chemistry

## Abstract

The escalating rate of fossil fuel combustion contributes to excessive CO_2_ emission and the resulting global climate change has drawn considerable attention. Therefore, tremendous efforts have been devoted to mitigate the CO_2_ accumulation in the atmosphere. Carbon capture and storage (CCS) strategy has been regarded as one of the promising options for controlling CO_2_ build-up. However, desorption and compression of CO_2_ need extra energy input. To circumvent this energy issue, carbon capture and utilization (CCU) strategy has been proposed whereby CO_2_ can be captured and *in situ* activated simultaneously to participate in the subsequent conversion under mild conditions, offering valuable compounds. As an alternative to CCS, the CCU has attracted much concern. Although various absorbents have been developed for the CCU strategy, the direct, *in situ* chemical conversion of the captured CO_2_ into valuable chemicals remains in its infancies compared with the gaseous CO_2_ conversion. This review summarizes the recent progress on CO_2_ capture and *in situ* catalytic transformation. The contents are introduced according to the absorbent types, in which different reaction type is involved and the transformation mechanism of the captured CO_2_ and the role of the absorbent in the conversion are especially elucidated. We hope this review can shed light on the transformation of the captured CO_2_ and arouse broad concern on the CCU strategy.

## Introduction

The demand for energy of the rapid industrialization results in large-scale combustion of fossil fuel, which causes excessive emissions of carbon dioxide. As the detrimental environmental impacts of CO_2_ have drawn considerable attention, various strategies have been developed to mitigate CO_2_ accumulation in the atmosphere, among which carbon capture and storage/sequestration (CCS) is considered as a promising CO_2_ reducing option (Alexander et al., 2015). Nowadays, a plethora of CO_2_ absorbents have been developed to facilitate CO_2_ capture and desorption. Nevertheless, the extensive energy needed in the absorbent regeneration and CO_2_ separation is not conducive to the implementation of CCS strategy.

In contrast to carbon sequestration, converting CO_2_ into valuable chemicals could be a sustainable option, which has been proposed by Ciamician as early as 1912 (Ciamician, 1912). In recent decades, CO_2_ conversion has attracted considerable concern and been intensively investigated (Rahman et al., 2017). However, in most processes for CO_2_ conversion, pure or high pressure CO_2_ is needed, implying that the CO_2_ from the atmosphere or industrial exhaust cannot be used as C_1_ source directly and thus the energy issue in CO_2_ capture and separation still remains.

**Scheme 1:**
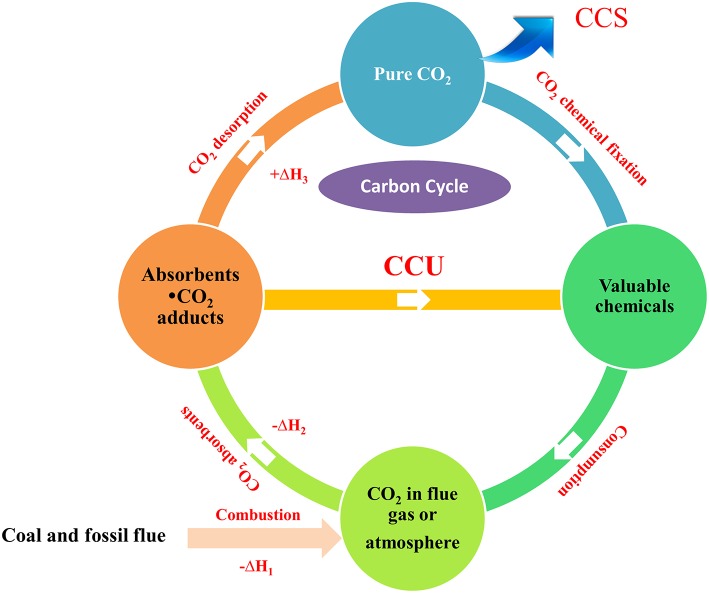
CCU strategy and its importance in carbon cycle.

To address the energy penalties associated with CCS strategy and realize the direct fixation of CO_2_ from the atmosphere or industrial exhaust, the CO_2_ capture and utilization (CCU) strategy, whereby the captured CO_2_ is used as a non-toxic, abundant, and sustainable feedstock to produce valuable organic compounds via chemical, electrochemical or photochemical reactions, was proposed and now is flourishing (Scheme 1). By now, both organic compounds and functional materials containing the bridging-carbonato metal complexes can be obtained from atmospheric CO_2_ using the CCU strategy (Yang et al., 2011; Liu et al., 2012; Massoud et al., 2015). Although realizing the attractive prospect of the CO_2_ capture and *in situ* conversion in the industry scale remains a challenge (Zhang and Lim, 2015), the emergence of efficient absorbents and the development of CO_2_ transformation will cast light on it. In continuation of our work on the conversion of the captured CO_2_ into value-added organic chemicals, this review summarized the recent progress on CO_2_ capture and *in situ* conversion into organic products.

**Scheme 2:**
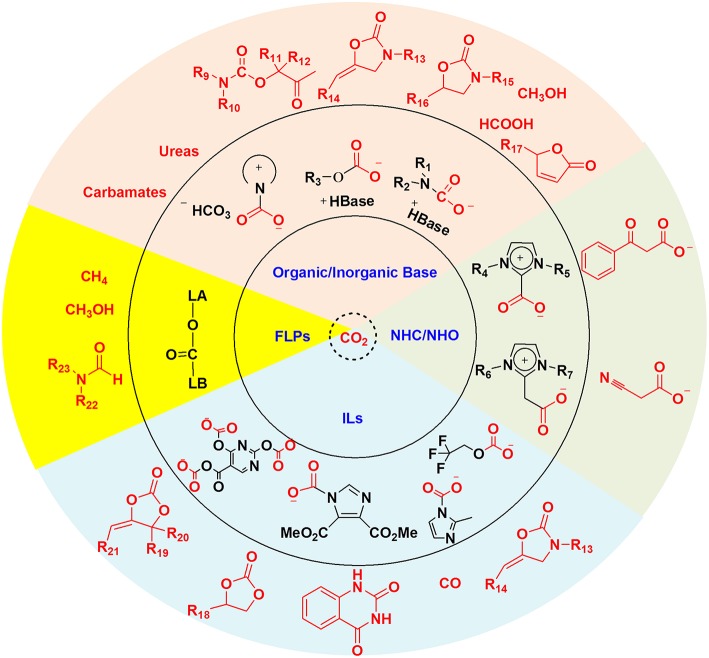
Absorbents, CO_2_ adducts, and the resulting valuable chemicals.

To realize the carbon capture and *in situ* conversion strategy, effective absorbents are always necessary. Ideally, the absorbents for CCU strategy should not only capture CO_2_, but also activate CO_2_ and even the substrate. Thus, the chemical transformation can proceed under mild conditions. Up to now, organic and inorganic bases, *N*-heterocyclic carbenes (NHCs) and *N*-heterocyclic olefins (NHOs), ionic liquids (ILs) and frustrated Lewis pairs (FLPs) have already been applied to CO_2_ capture and *in situ* conversion. A plethora of valuable organic chemicals have been obtained through the CCU strategy as shown in Scheme 2.

## Inorganic/Organic Bases

Due to the electrophilicity of carbon atom in CO_2_, the organic and inorganic bases containing strong nucleophilic atom have been widely used in CO_2_ trapping, where the base can interact with CO_2_ directly or function as a proton acceptor. The resulting CO_2_ capture products i.e., CO_2_ adducts have been employed for subsequent synthesis of various valuable chemicals.

Considering the transformations of the captured CO_2_ derived from primary and secondary amines and amino alcohols to isocyanates, carbamates, ureas, and oxazolidinones have been concerned by several excellent review papers (Hampe and Rudkevich, 2003; Chaturvedi and Ray, 2006; Yang et al., 2012; Tamura et al., 2014; Wang et al., 2017a,b), here we focus on the transcarboxylation effect and other transformations of the captured CO_2_, namely CO_2_ derivatives.

### Synthesis of Carbamates and Ureas

In the synthesis of carbamates, the aprotic organic bases can function as CO_2_ absorbents and transcarboxylation agents. The initial attempt was made by Rossi group, in which CO_2_ is trapped by a methanol solution of commercially available tetraethylammonium hydroxide. The resulting tetraethylammonium hydrogen carbonate can be used as a surrogate of CO_2_ in the synthesis of carbamate. Meanwhile, the presence of tetraethylammonium ion as counterion increases the nucleophilicity of carbamate anion (Inesi et al., 1998).

**Scheme 3:**
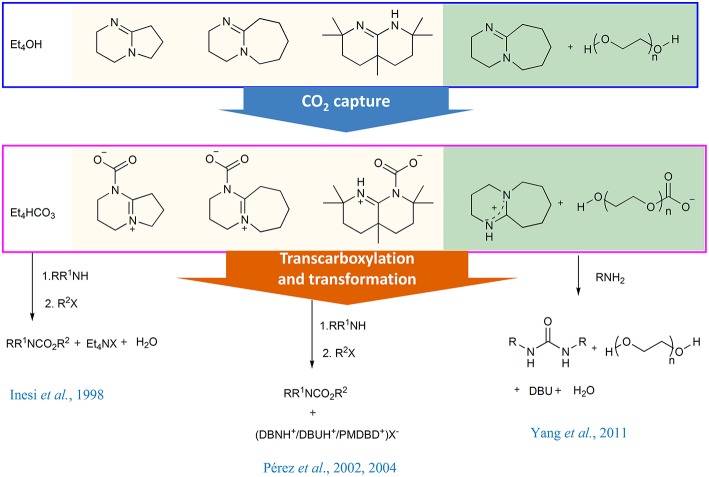
CO_2_ capture and transcarboxylation in the synthesis of carbamates and ureas.

Soon after, Franco group has successfully identified the DBU-CO_2_ complex via reacting CO_2_ with DBU (1,8-Diazabicyclo[5.4.0]undec-7-ene) in anhydrous acetonitrile, implying that DBU can be used as CO_2_ trap reagent (Pérez et al., 2002). Moreover, the resulting reactive DBU-CO_2_ adduct can be utilized as transcarboxylating reagent for synthesis of *N*-alkyl carbamates. Later, the same group revealed the activation capacity of CO_2_ by other bicyclic amidines and observed the inverse relation between the thermal stability and the transcarboxylating activity for the amidine-CO_2_ adducts (Scheme 3) (Pérez et al., 2004), which is the first time to investigate the activation ability of organic bases to CO_2_.

**Scheme 4:**
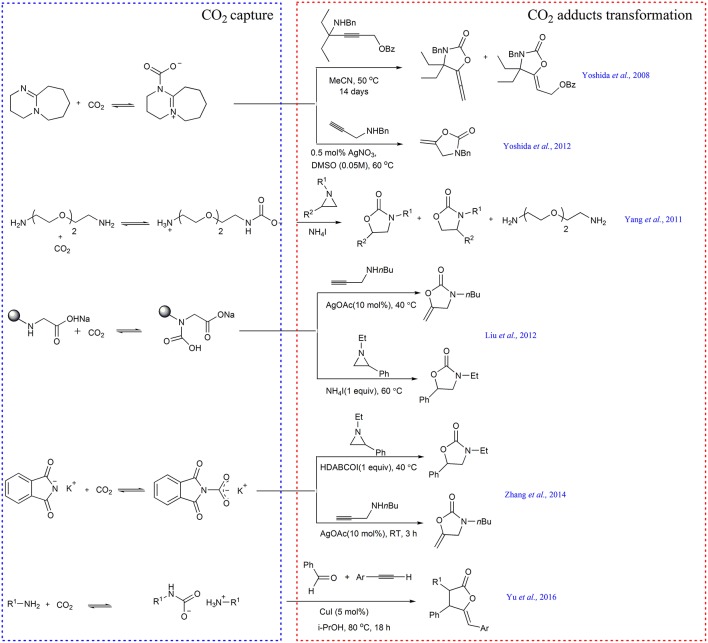
CO_2_ capture and transformation to oxazolidinones.

The combination of organic base and alcohol is an efficient CO_2_ capture system and the absorbed CO_2_ can be *in situ* transformed. The prototypical example is the polyethylene glycol (PEG)/superbase system developed by our group in 2011 (Yang et al., 2011). In the capture step, the superbase is used as a proton acceptor and almost equimolar CO_2_ per mole superbase can be absorbed (Scheme 4). The resulting liquid amidinium carbonate can directly react with *n*-butylamine at 110°C to afford dibutyl urea in almost quantitative yield (96%) without any other additives. This protocol can be used in the synthesis of other symmetrical urea derivatives.

In the above examples, the captured CO_2_ in the transcarboxylating agents can be regarded as the activated CO_2_ because the linear structure of CO_2_ is converted to bent structure, which is more liable to nucleophilic attack.

### Synthesis of Oxazolidinones

The “CO_2_ absorption and subsequent transcarboxylation” triggers the research on CO_2_ capture and *in situ* transformation. Several years later, M. Yoshida and coworkers use DBU to enrich and activate CO_2_ in air and perform the first example of directly transforming atmospheric CO_2_ into the substituted 5-vinylideneoxazolidin-2-ones using propargylic substrate 4-(benzylamino)-2-butynyl carbonates or benzoates as a substrate (Scheme 4) (Yoshida et al., 2008). In their follow-up work, they further improve the reaction efficiency by utilizing AgNO_3_ as catalyst and propargylic amines as substrates (Scheme 4) (Yoshida et al., 2012).

Inspired by these works, our group designs a series of novel CO_2_ capture and activation systems. For example, by employing ammonium iodide as catalyst, the cycloaddition reaction of various aziridines with the captured CO_2_ by NH_2_PEG_150_NH_2_ gives rise to oxazolidinones at 40°C in >94% yield and selectivity (Scheme 4) (Yang et al., 2011).

Soon after, we report the first example of steric-hindrance-controlled CO_2_ absorption, where the sodium *N*-alkylglycinates and *N*-alkylalaninates dissolved in PEG_150_ are used to capture CO_2_, generating the carbamic acid rather than the ammonium carbamate (Liu et al., 2012). *N*-isopropylglycinate is found to be the best absorbent for the rapid and reversible capture of almost equimolar CO_2_. Crucially, the captured CO_2_ can be activated simultaneously and the resulting carbamic acid can react with either aziridine or propargyl amine to afford oxazolidinones in the presence of NH_4_I and AgOAc as a catalyst, respectively (Scheme 4).

Motivated by these results, we further develop potassium phthalimide as absorbent to realize equimolar CO_2_ capture in PEG_150_. Moreover, the obtained product can be used as *in situ* transcarboxylating reagent to synthesize oxazolidinone derivatives (Scheme 4) (Zhang et al., 2014).

Recently, Hu group subtly designs a CCU example (Yu et al., 2016), in which carbamate salts generated from CO_2_ and primary amines are used as substrates. The captured CO_2_ not only acts as a reactant but also acts as a protecting reagent for the amine to avoid poisoning of the copper catalyst. By using 5 mol% of CuI as catalyst, carbamate salts can react with aromatic aldehydes and aromatic terminal alkynes, affording the important oxazolidin-2-ones (Scheme 4).

### Synthesis of β-Oxopropylcarbamates

**Scheme 5:**
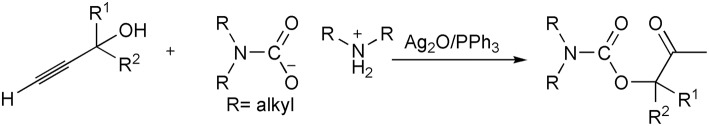
Synthesis of β-oxopropylcarbamates from propargylic alcohols and ammonium carbamates.

Based on these inspiring results, our group uses ammonium carbamates as surrogates of carbon dioxide and secondary amines in the three-component synthesis of β-oxopropylcarbamates from propargylic alcohols, secondary amines, and CO_2_. Catalyzed by silver (I) catalyst, ammonium carbamates can react with propargylic alcohols to generate β-oxopropylcarbamates under atmospheric pressure (Scheme 5) (Song et al., 2015). In this example, the substitution of pure CO_2_ with the captured CO_2_ can facilitate the reaction running at atmospheric pressures with a broad substrate and reaction application scope. Furthermore, the solid ammonium carbamates are easier to handle and quantify than the volatile amines and gaseous CO_2_.

The transcarboxylation is an important transformation strategy for the CO_2_ adducts to valuable chemicals. However, for the CO_2_ adducts formed by base and CO_2_, the transcarboxylation is still limited to the substrates including amines, propargylamines and aziridines. Therefore, novel CO_2_ absorbents and extended substrates are expected to facilitate the application of CO_2_ adducts as transcarboxylation reagent in CCU strategy. Besides transcarboxylation, the integral transformation of CO_2_ capture products is another attractive option in CCU strategy, wherein the ammonium carbamates derived from CO_2_ and amines is a promising raw material. Nevertheless, the integral transformation of ammonium carbamates to valuable chemicals remains sporadic and underexplored. Hopefully, more conversion protocols of ammonium carbamates can be designed based on the reactivity of amine and CO_2_.

### CO_2_ Capture and *in situ* Hydrogenation

CO_2_ hydrogenation is widely investigated in the CCU strategy because the basic absorbent can react with the resulting formic acid to form formate, thus overcomes the thermodynamic limitation in the hydrogenation of CO_2_. In the researches on CO_2_ capture and *in situ* hydrogenation, both metal-based homogeneous catalysts (containing Rh-, Ru-, and Fe-based catalysts) and heterogeneous catalysts have been investigated.

#### Hydrogenation Using Rh-Based Catalysts

**Scheme 6:**
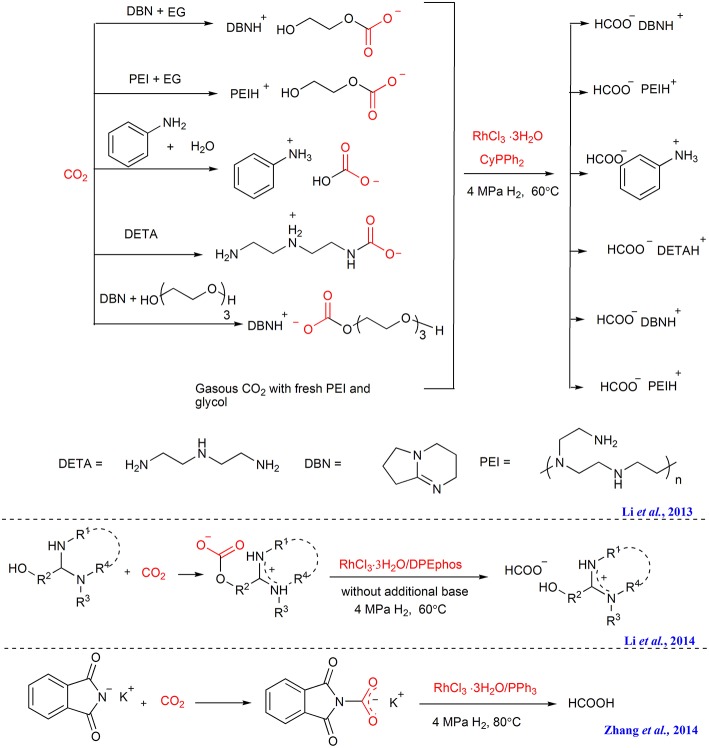
CO_2_ capture and Rh promoted CO_2_ adducts hydrogenation.

The first example of CO_2_ capture and *in situ* hydrogenation is reported by our group in 2013, in which polyethyleneimine 600 (PEI_600_) or the combination of PEI_600_ and ethylene glycol is developed to absorb gaseous CO_2_, affording the PEI-CO_2_ or ethylene glycol-CO_2_ adducts. With RhCl_3_·3H_2_O/CyPPh_2_ as catalyst, the captured CO_2_ can be *in situ* transformed to formate (Scheme 6) (Li et al., 2013). Furthermore, direct hydrogenation of ammonium carbamate derived from CO_2_, e.g., DETA^+^CO_2_^−^ (DETA = diethylenetriamine), ammonium carbonates such as [DBNH] [OCO_2_(C_2_H_4_O)_3_H], [DBNH] [OCO_2_CH_2_OH] (DBN = 1,5-diaza bicycle[4.3.0]non-5-ene), is also successfully performed facilitated by this Rh-based catalyst. Notably, a higher reaction rate and better results can be achieved when using the captured CO_2_ in the form of ammonium carbonates as feedstock than using equivalent free gaseous CO_2_ or ammonium carbamate, implying CO_2_ activation upon capture with DBN/PEI and glycol.

After that, we further design a tunable ethoxyl-functionalized amidine to absorb CO_2_ in order to avoid the use of volatile proton donor (Li et al., 2014). As an activated form of CO_2_, the captured CO_2_ in the form of zwitterionic amidinium carbonate is further hydrogenated to formate employing RhCl_3_/DPEphos as the catalyst (Scheme 6). In the same time, we find that the CO_2_ capture product of potassium phthalimide in PEG_150_, can also be *in situ* hydrogenated to formic acid catalyzed by RhCl_3_·3H_2_O/CyPPh_2_ (Scheme 6) (Zhang et al., 2014).

#### Hydrogenation Using Ru- and Fe-Based Catalysts

**Scheme 7:**
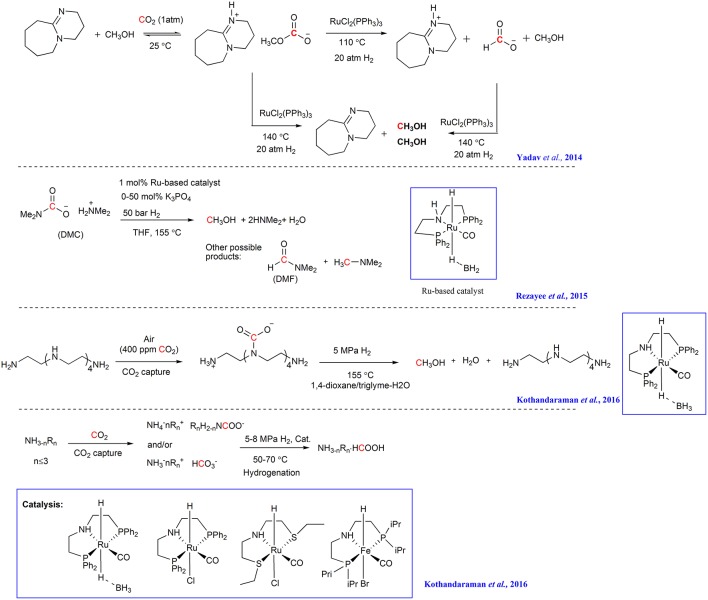
CO_2_ capture and Ru promoted CO_2_ adducts hydrogenation.

Ru-based catalysts are also promising candidates for the hydrogenation of captured CO_2_. In the study of Yadav et al., CO_2_ is captured by DBU and an alcohol to form the alkyl carbonate ionic liquid and the resulting alkyl carbonate is hydrogenated into [DBUH^+^] formate and methyl formate facilitated by RuCl_2_(PPh_3_)_3_ (Scheme 7) (Yadav et al., 2014). Although the reactive species (i.e., alkyl carbonates or CO_2_) cannot be identified at this stage, this result indicates that alkyl carbonates may be a substrate for hydrogenation, in addition to free CO_2_.

In 2015, Sanford group combined CO_2_ capture to form a carbamate salt with hydrogenation to generate CH_3_OH (Rezayee et al., 2015). In their study, NHMe_2_ is used to capture CO_2_ and a homogeneous Ru-based catalyst is used to facilitate the hydrogenation of the captured CO_2_ to a mixture of DMF and CH_3_OH (Scheme 7). Although the formation of carbamate salt can decrease the eletrophilicity of CO_2_, causing the captured CO_2_ difficult to hydrogenate, the existence of the equilibrium between DMC (Dimethylammonium dimethylcarbamate) and CO_2_ allows the release of CO_2_ possible, thus promoting the CO_2_ hydrogenation.

By employing pentaethylenehexamine (PEHA) as CO_2_ absorbent and Ru-based complexes as catalyst, Olah and Surya Prakash group develops a process that combines CO_2_ capture and the following hydrogenation in an ethereal solvent for the production of MeOH (Kothandaraman et al., 2016a). CO_2_ from air can be captured by an aqueous solution of PEHA and up to 61% yield of MeOH can be obtained in the triglyme/H_2_O mixtures at 155°C in the following hydrogenation (Scheme 7). The resulting MeOH can be easily separated by simple distillation from the reaction mixture.

Later, the same group captures CO_2_ with aqueous amine solution and then *in situ* hydrogenates the resulting ammonium bicarbonate/carbonate utilizing Ru- and Fe-based pincer complexes in a biphasic solvent system (water/Me-THF) (Kothandaraman et al., 2016b). The superbases (DABCO, TMG, and DBU) shows to be efficient for both CO_2_ capture and hydrogenation with more than 90% yield of formate under moderate reaction conditions (50 bar H_2_ at 55°C) (Scheme 7). The biphasic system features easy separation of product and catalyst and the catalyst can be reused for at least five cycles.

#### Hydrogenation Using Heterogeneous Catalysts

**Scheme 8:**
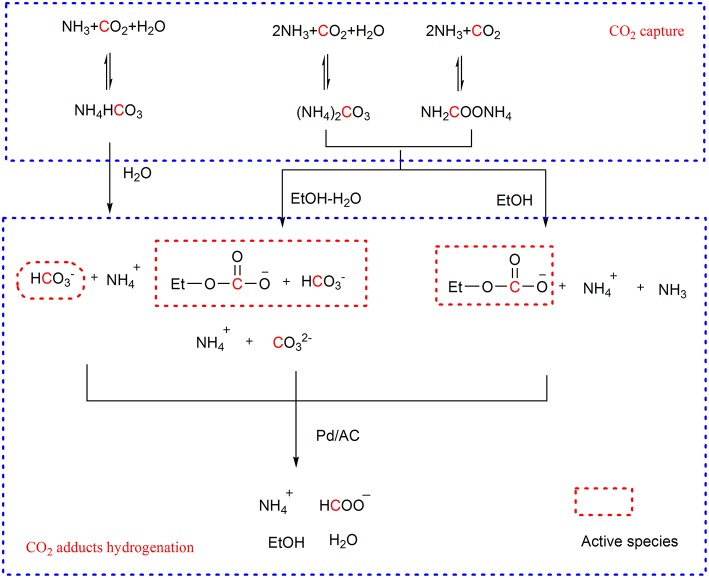
CO_2_ capture and Pd/AC facilitated CO_2_ adducts hydrogenation.

Besides homogeneous catalysts, the heterogeneous catalysts were also used in the hydrogenation of CO_2_ capture products. For example, H. Lin group applies Pd/AC catalyst to the hydrogenation of CO_2_ capture products originated from ammonia. In the hydrogenation step, the dependence of the activity of CO_2_ capture products on the solvent is observed (Su et al., 2015a,b). For example, the ammonium bicarbonate in water and ammonium carbamate in 70 wt% ethanol-water solution can offer more than 90% yield of formate under high H_2_ pressure (5.52 and 2.75 MPa, respectively) at 20°C. The ammonium carbonate presents similar activity with ammonium carbamate. Identification of the species in the reactant solutions suggests the bicarbonate ion and ethyl carbonate ion, instead of the carbamate ion, are the activation forms of CO_2_ in the hydrogenation (Scheme 8). Coincidently, Enthaler finds that sodium bicarbonate in methanol can be hydrogenated to sodium formate catalyzed by the nickel hydride complex while CO_2_ cannot be hydrogenated in the identical conditions, which further confirms the activity of the captured CO_2_ in hydrogenation (Enthaler et al., 2015).

**Scheme 9:**
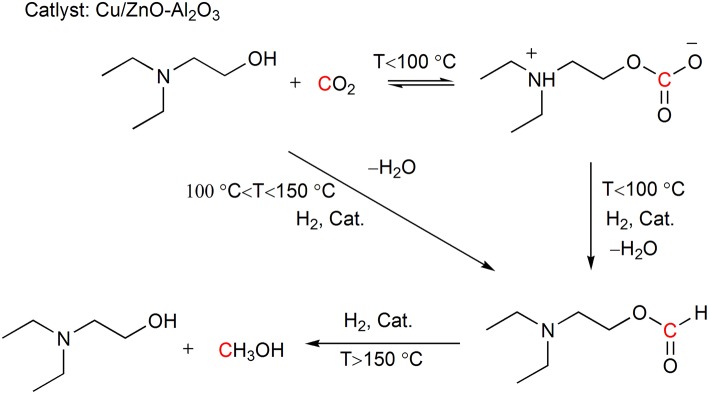
Cu/ZnO-Al_2_O_3_ promoted CO_2_ adducts hydrogenation.

Mertens and coworkers report the *in situ* hydrogenation of the captured CO_2_ using Cu/ZnO-Al_2_O_3_ as catalyst under retrieval of the CO_2_ capture reagent N,N-diethylethanolamine (DEEA) (Reller et al., 2014). In the reaction, DEEA can also function as a trapping reagent for the resulting formic acid and drives the hydrogenation forward. The authors find that the generation of the products 2-diethylaminoethylformate and methanol can be regulated by the reaction temperature (Scheme 9). The combination of CO_2_ capture and hydrogenation realizes the energy integration by using the reaction heat of CO_2_ hydrogenation in the energy demanding CO_2_ stripping process.

In addition to the liquid absorption system, the alkali metal and alkali earth metal based solid CO_2_ adsorbents are also developed (Li et al., 2010, 2011; Lee et al., 2011) and applied in the CCU strategy recently. Duyar et al. design a series of novel dual function materials (DFM) consisting of the catalyst and adsorbent components to couple the endothermic CO_2_ desorption step with the exothermic hydrogenation of CO_2_ (Duyara et al., 2016). The results show that DFM with the composition of 5% Ru 10% K_2_CO_3_/Al_2_O_3_ and 5% Ru 10% Na_2_CO_3_/Al_2_O_3_ have a methanation capacity of 0.91 and 1.05 g-mol/kg DFM, respectively. Similarly, A. Urakawa group develops the catalyst consisting of earth-abundant chemical elements (FeCrCu/K/MgO–Al_2_O_3_), which can trap CO_2_ from fuel gas in the form of surface carbonates and subsequently hydrogenated the adsorbed CO_2_ to CO (Bobadilla et al., 2016). Accordingly, these DFMs are identified as promising candidates for CO_2_ capture and direct utilizations.

Hydrogenation of captured CO_2_ to energy chemicals can facilitate turning hydrogen gas to liquid fuel as well as realize carbon cycling. Albeit the hydrogenation of captured CO_2_ has been extensively investigated and various capture reagents and catalysts have been developed, the identification of CO_2_ activation forms is still controversial. For example, the CO_2_ capture products alkyl carbonate ammonium salts are considered as the activated CO_2_ species (Li et al., 2013, 2014; Su et al., 2015a,b). However, the results of Jessop group show that [DBUH][OC(O)OMe] salt is less active than free CO_2_ when using RuCl(O_2_CMe)(PMe_3_)_4_ as catalyst in MeOH solution (Munshi et al., 2002). Thus, the relationship between the activity of CO_2_ capture products and the catalyst is still underdeveloped.

## *N*-heterocyclic Carbenes and *N*-heterocyclic Olefins

**Scheme 10:**
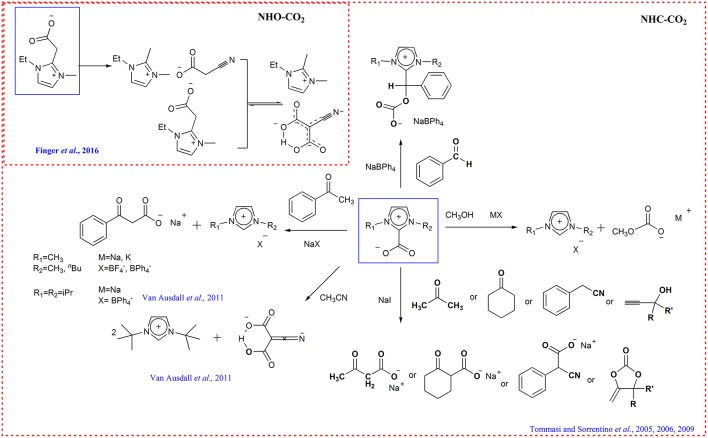
Transcarboxylation of NHC-CO_2_ and NHO-CO_2_ adducts.

It has been verified that *N*-heterocyclic carbenes and *N*-heterocyclic olefins can react with CO_2_, forming the CO_2_ adduct which can be used as “all-in-one” carboxylating agent (Zhou et al., 2008; Kelemen et al., 2014; Dong et al., 2015; Talapaneni et al., 2015; Finger et al., 2016; Saptal and Bhanage, 2016). For example, Tommasi group shows the CO_2_ adduct 1-butyl-3-methylimidazolium-2-carboxylate and 1,3-dimethylimidazolium-2-carboxylate behaves as active CO_2_-carriers and reacted with CH_3_OH and acetophenone for the synthesis of methylcarbonate and benzoylacetate. The other organic compounds with active hydrogen (acetone, cyclohexanone, benzylcyanide, and propargyl alcohols) can also be carboxylated with these CO_2_-transfer agents for the synthesis of carboxylates of pharmaceutical interest (Tommasi and Sorrentino, 2005, 2006, 2009) (Scheme 10). Similarly, the transcarboxylation of IPrCO_2_ (1,3-bis(2,6-diisopropylphenyl)-imidazolum-2-carboxylate) to acetophenone with NaBPh_4_ to yield sodium benzoylacetate and direct dicarboxylation of MeCN using I^*t*^BuCO_2_ (1,3-bis(tert-butyl)-imidazolium-2-carboxylate) are also reported (Van Ausdall et al., 2011) (Scheme 10).

The transcarboxylation capacity of NHOs-CO_2_ adducts has also been verified by 1-ethyl-3-methyl-imidazolium-2-methylenecarboxylate through realizing the C-C coupling of CO_2_ and MeCN (Scheme 10) (Finger et al., 2016). In this transcarboxylation process, the basicity of NHOs should be strong enough to abstract proton from the CH acid.

As highly efficient carboxylating agents, the NHC-CO_2_ and NHO-CO_2_ complexes can be easily obtained by reacting NHCs or NHOs with atmospheric CO_2_. However, instead of serving as absorbent, NHCs and NHOs are usually used as catalysts to promote the conversion of pure CO_2_ by forming transient NHC-CO_2_ and NHO-CO_2_ complexes (Kayaki et al., 2009; Zhou et al., 2017). The reason is that NHCs and NHOs are sensitive to air and moisture thus they cannot be used as absorbents for CO_2_ in air and industry exhaust. Nowadays, it is found that the imidazolium ionic liquids containing basic anion can absorb CO_2_, producing imidazolium carboxylates (Gurau et al., 2011; Wang and Wang, 2016). Considering the imidazolium ionic liquids are stable to air and moisture, it opens a new way for the utilization of NHC-CO_2_ and NHO-CO_2_ complexes in CCU strategy.

## Ionic Liquids (ILs)

Ionic liquids (ILs) offer a new opportunity for developing novel CO_2_ capture reagents (Huang and Rüther, 2009; Gurkan et al., 2010; Wang et al., 2011; Yang and He, 2014). Especially, the active site-containing ionic liquids can trap and activate CO_2_ through chemical absorption. Besides, IL can also function as catalyst in CO_2_ transformation (Lang et al., 2016; Zhang et al., 2017; Xia et al., 2018). Therefore, it is promising to combine the multiple roles of ILs in CCU strategy. Up to now, cyclocarbonates, oxazolidinones and quinazoline-2,4-(1H,3H)-diones have been synthesized using ILs as CO_2_ absorbents and catalysts.

**Scheme 11:**
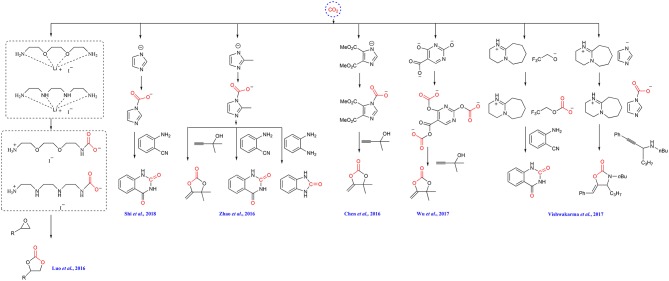
ILs used in CO_2_ capture and conversion.

Wang group performs a series of investigation on ILs-based CO_2_ capture and conversion. For example, they design bifunctionalized ionic liquids to capture and simultaneously fix CO_2_ in the simulation of fuel gas to cyclic carbonates (Scheme 11) (Luo et al., 2016). The cation can capture CO_2_ and the anion I^−^ can activate the substrate to facilitate CO_2_ insertion. In the presence of a small amount of water, the yield of product can be improved, making this reaction more applicable to industrial exhaust.

Later, the same group finds that the basicity of anion of ILs is very important for CO_2_ capture and transformation. A hydroxyl functionalized aprotic ionic liquid shows high efficiency in synthesis of quinazoline-2,4(1H,3H) -diones from atmospheric CO_2_. The captured CO_2_ instead of atmospheric CO_2_ is also used and only 13% yield is obtained, being ascribed to the strong interaction between [Im]^−^ and CO_2_ (Scheme 11) (Shi et al., 2018). They also demonstrates the feasibility of using captured CO_2_ as starting material in their another report, where the CO_2_ captured by azole-type anion [DEIm]^−^ renders a high yield of alkylidene carbonates in the carboxylative cyclization of propargyl alcohol due to the weak interaction between CO_2_ and the anion (Scheme 11) (Chen et al., 2016).

Liu group reports that azole-anion-based ILs with the [Bu_4_P]^+^ cation can capture CO_2_. With appropriate substrates, the forming carbamate intermediates can be transformed into the α-alkylidenecyclic carbonate, quinazoline-2,4(1H,3H)-diones and benzimidazolone without other catalysts (Scheme 11) (Zhao et al., 2016). Later, the same group reveals that a series of tetrabutylphosphonium ([Bu_4_P]^+^)-based ILs with multiple-site for CO_2_ capture and activation in their anions can be used in CO_2_ capture and conversion, wherein the IL [Bu_4_P]_3_[2,4-OPym-5-Ac] shows the optimal performance in preparation of α-alkylidene cyclic carbonates from propargylic alcohol substrate (Wu et al., 2017). The resulting polycarbonates derived from CO_2_ and the anion is proved to be the key intermediate in this reaction (Scheme 11).

The ILs [HDBU][MIm] and [HDBU][TFE] can capture CO_2_ and also show catalytic activity to the reaction of CO_2_ and propargylic amines for the synthesis of 2-oxazolidinones and the reaction of CO_2_ with 2-aminobenzonitrile derivatives to synthesis quinazoline-2,4-(1H,3H)-diones. By enhancing the mass transfer with gas-liquid laminar flow continuous-flow microreactor, the simultaneous capture and fixation CO_2_ to 2-oxazolidinones and quinazoline-2,4-(1H,3H)-diones is realized (Scheme 11) (Vishwakarma et al., 2017).

Due to the dual function as CO_2_ absorbents and conversion catalysts, ionic liquids can realize the transformation of captured CO_2_ with several kinds of substrates. Furthermore, the non-volatility characteristic of ionic liquids can facilitate product separation. Thus, the ionic liquids are considered as promising absorbents for the CCU process.

## Frustrated Lewis Pairs (FLPs)

**Scheme 12:**
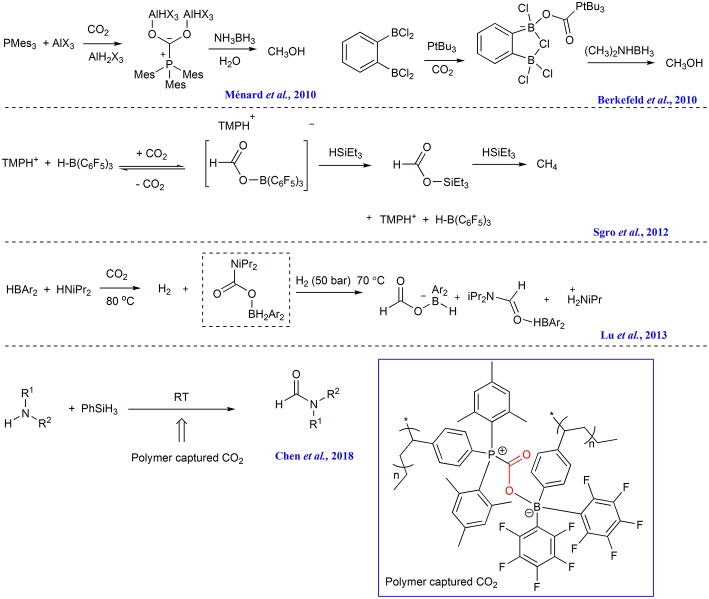
FLPs employed in CO_2_ capture and transformation.

The CO_2_ capture capacity of FLPs has been reported soon after the FLPs concept was in 2006 proposed (Welch et al., 2006; Momming et al., 2009; Travis et al., 2013; Weicker and Stephan, 2015; Wolff et al., 2016). As early as 2010, Stephan group revealed that 1:2 mixtures of PMes_3_/AlX_3_ (X = Cl or Br) in bromobenzene can react with CO_2_, forming CO_2_ adduct which can be converted to CH_3_OH with ammonia borane as reductant (Scheme 12) (Ménard and Stephan, 2010). In the same year, Piers group found that CO_2_ captured by FLP consisting of 2,2,6,6-tetramethylpiperidine (TMP) and B(C_6_F_5_)_3_ can be reduced to methane with triethylsilane (Scheme 12) (Berkefeld et al., 2010). Soon, it is found that the FLP composed by bis-borane 1,2-C_6_H_4_(BCl_2_)_2_ and P*t*Bu_3_ can capture CO_2_ and the forming capture product can be reduced to methanol by reductant such as amine-borane Me_2_NHBH_3_ or [C_5_H_6_Me_4_NH_2_][HB(C_6_F_5_)_2_(C_7_H_11_)] (Scheme 12) (Sgro et al., 2012). Wang group reports the FLP comprising of bis(2,4,6-tris(trifluoromethyl)phenyl)borane and a secondary amine (such as HN*i*Pr_2_ or HNEt_2_) readily reacts with CO_2_ at 80°C, affording carbamate boryl esters which can function as an intramolecular FLP to activate H_2_, affording ammonium borylformate salt and formamide adducts (Scheme 12) (Lu et al., 2013).

Recently, Yan group incorporates FLP acceptor and donor into the styrene-based monomers, respectively to prepare two diblock copolymers consisting of the complementary FLP blocks and common polystyrene block. These two diblock copolymers can bind CO_2_, forming nanoparticle. The nanoparticle is then used as CO_2_ reservoir and catalyst to facilitate the formylation of amines with phenylsilane (Scheme 12) (Chen et al., 2018).

The CO_2_ capture and H_2_ activation capacity makes FLPs attractive for CCU strategy, especially for the hydrogenation of captured CO_2_. However, in the current study, the FLPs promoted CO_2_ hydrogenation encounters difficulty in FLPs regeneration (Ashley et al., 2009). By now, the FLPs are merely used as CO_2_ capture reagents and reductants are still needed. Thus, the hydrogen activation ability of FLPs hasn't been utilized. Recently, the breakthrough is made by Jazzar and Bertrand group. By combining the copper catalyst and Lewis pair, hydrogenation of carbon dioxide into formate is realized (Romero et al., 2018). Latter, X. Hu and Y. Wu group reports the first catalytic hydrogenation process of CO_2_ to formate using transition metal free catalyst (B(C_6_F_5_)_3_/M_2_CO_3_, M = Na, K, and Cs) (Zhao et al., 2019). These results open new vistas in the field of FLPs facilitated CO_2_ capture and hydrogenation.

## Catalyst design for direct conversion of diluted CO_2_

**Scheme 13:**
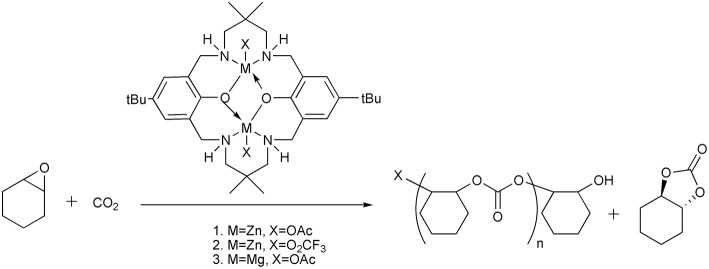
Homogeneous dinuclear Zn or Mg catalysts used for the production of poly(cyclohexene carbonate).

In addition to the CO_2_ capture and transformation strategy, there are also examples that the CO_2_ from waste streams can be directly converted by designing catalysts that tolerate to the contaminants in the waste streams such as exogenous water, nitrogen, SO_2_, amine etc. For example, Williams et al. reports the synthesis of poly(cyclohexylene carbonate) using the power station generated CO_2_ facilitated by the homogeneous dinuclear Zn or Mg catalysts, which is stable in the presence of contaminants from gas streams (Chapman et al., 2015) (Scheme 13). D'Elia and Basset group develops the combination of early transition metal halides (Y, Sc, Zr) and TBAB to quantitatively convert CO_2_ from diluted streams and produce cyclic organic carbonates (Barthel et al., 2016). The features of metal-organic frameworks (MOFs) to selectively capture and catalyze CO_2_ conversion make them a new type of platform for diluted CO_2_ transformation. Recently, Hong group design and synthesize an acid-base resistant Cu(II)-MOF which can convert CO_2_ from simulated post-combustion flue gas into corresponding cyclic carbonates (Liang et al., 2017). In direct conversion of diluted CO_2_, the stability of the catalysts to the contaminants in the gas streams is crucial to the success of the process.

## Conclusion and Outlook

The past 10 years have witnessed great advances in CO_2_ capture and *in situ* conversion. Different CO_2_ absorbents including inorganic and organic bases, NHCs, and NHOs, ILs, FLPs and polymeric functional materials have been employed in this field. As an emerging field, much effort is still desired to explore the potential conversion of the captured CO_2_.

Considering amines and CO_2_ can be involved in diverse reactions, it is hoped that the conversion of ammonia carbamate can be further extended to other valuable products besides isocyanates, carbamates and ureas.

On the other hand, although the hydrogenation of CO_2_ captured by inorganic/organic bases and the combination of base and alcohol has been investigated, the hydrogenation of CO_2_ captured by ILs and/or FLPs remains sporadic and underexplored. In light of the successful electrochemical reduction of IL-captured CO_2_ and hydrogenation of CO_2_ in ILs (Zhang et al., 2008, 2009; Wesselbaum et al., 2012; Scott et al., 2017), it is reasonable to conclude that the captured CO_2_ by IL can be hydrogenated by designing appropriate catalyst. Besides, the breakthrough in FLP-mediated CO_2_ hydrogenation opens new possibilities for FLP-based CO_2_ capture and transformation (Romero et al., 2018; Zhao et al., 2019).

Another important transformation pathway for CO_2_ adducts derived from reacting with amines, NHCs, NHOs and ILs is transcarboxylation reaction in the synthesis of cyclocarbonates, oxazolidinones and quinazoline-2,4-(1H,3H)-diones. It is foreseeable that the application of these transcarboxylating reagents will be investigated continuously with the emergence of new CO_2_ conversion reactions.

Although a plethora of CO_2_ absorbents has been developed and valuable products can be obtained from the resulting CO_2_ adducts by different strategy, however, the cost and feasibility must be considered from the viewpoint of industrial application. With this in mind, amines and ionic liquids are considered as promising absorbents for the CCU strategy. For amine absorbents, their features of high absorbing capacity and comerical availability are attractive for industrial application. Besides, the CO_2_ adducts derived from amines and CO_2_ are non-volatile liquids or solids, which can be used as starting materials instead of the volatile amines and CO_2_ gas to develop the gas free progress. For ionic liquid- based CO_2_ absorbents, the acceptable cost, tunable structure as well as their high CO_2_ capture capacity make them competent to the commercial CCU process. Moreover, the ionic liquids are nonvolatile and the corrosion of equipment can be avoided by subtly design the structure of ionic liquids, all of which can facilitate the industry operability.

In summary, the CO_2_ capture and *in situ* catalytic transformation is still in its infancy. We hope this review can inspire the extensive research on CO_2_ capture and *in situ* transformation, which will benefit for the design of efficient CO_2_ capture and utilization system (CCU) and realize the valorization of diluted CO_2_ in waste gas streams or directly from the atmosphere.

## Author Contributions

All authors contributed for the writing of the manuscript. L-NH designed this proposal and determined the contents. H-RL wrote the Abstract, Introduction, Conclusion, and Outlook parts. H-CF wrote the Inorganic/organic bases and Ionic liquids parts. FY wrote *N*-heterocyclic carbenes and *N*-heterocyclic olefins and frustrated lewis pairs parts. H-RL and L-NH revised the manuscript.

### Conflict of Interest Statement

The authors declare that the research was conducted in the absence of any commercial or financial relationships that could be construed as a potential conflict of interest.
